# Role of molecular signature to differentiate second primary lung cancer from metastasis in a patient with squamous cell carcinoma of oral cavity

**DOI:** 10.1002/cnr2.1363

**Published:** 2021-06-23

**Authors:** Emrullah Yilmaz, Gregory N. Gan, Thomas M. Schroeder, Andrew Cowan, Nancy Joste

**Affiliations:** ^1^ Department of Hematology and Medical Oncology Cleveland Clinic Cleveland Ohio USA; ^2^ Department of Radiation Oncology University of Kansas Medical Center Kansas City Kansas USA; ^3^ University of New Mexico Comprehensive Cancer Center University of New Mexico Albuquerque New Mexico USA; ^4^ Department of Pathology University of New Mexico Albuquerque New Mexico USA

**Keywords:** head and neck cancer, lung cancer, lung metastasis, molecular signature

## Abstract

**Background:**

Lung is the most common site of distant metastasis for patients with head and neck squamous cell carcinoma (HNSCC). However, differentiating second primary lung cancers from metastasis may be difficult for p16 negative HNSCC.

**Case:**

We describe a case of oral cavity squamous cell carcinoma (SCC) who was found to have lung nodule and hilar lymphadenopathy (LAD) after surgery and radiation therapy. Hilar node was consistent with SCC however, it was difficult to differentiate second primary lung cancer and metastasis from oral cavity SCC.

Next‐generation sequencing was done for the primary oral cavity and the hilar node. Both samples had the same type of *TP53* mutation and variants of unknown significance suggesting metastatic HNSCC. He was treated with a chemotherapy regimen for metastatic HNSCC.

**Conclusion:**

Molecular studies can help to differentiate metastasis from second primary lung cancers for p16 negative HNSCC.

## 1 INTRODUCTION

Head and neck squamous cell carcinoma (HNSCC) patients are at risk for developing both lung metastasis and second primary lung squamous cell carcinoma.^1^, ^2^ P16 immunohistochemistry (IHC) can help to differentiate the metastasis vs the second primary if the primary HNSCC is human papilloma virus (HPV) related.^3^ However, diagnosis is more challenging for HPV‐unrelated, p16 negative HNSCC. The role of the IHC is limited to distinguish second primary lung SCC. Thyroid transcription factor 1 (TTF‐1) can be positive in 7%‐10% of primary lung carcinoma, and not found in head and neck cancer.^4^ An algorithm using cytokeratin 19 (CK19), matrix metallopeptidase 3 (MMP3), and peptidase inhibitor 3 (PI3) IHC was suggested to help differentiate metastasis from second lung primary SCC.^5^ Loss of heterozygosity and *TP53* mutations were found to be stable during tumor progression in HNSCC.^6^ Therefore LOH and *TP53* status were suggested as markers to diagnose metastasis in retrospective studies.^6^, ^7^, ^8^ In the era of molecular studies, we suggest that molecular profiling can help to differentiate metastasis vs the second primary for HPV negative cancers for these cancers. Here, we present the use of comprehensive molecular profiling in a patient with squamous cell carcinoma (SCC) of the oral cavity.

## 2 CASE REPORT

**FIGURE 1:**
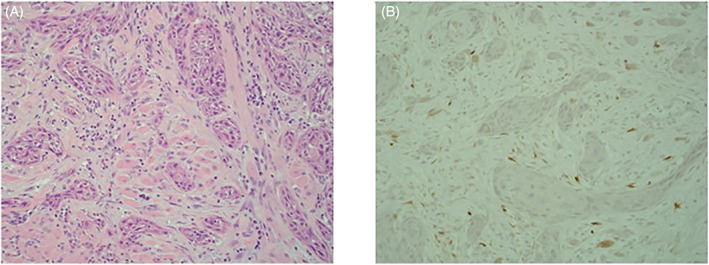
Invasive squamous cell carcinoma involving the tongue. A, infiltrating nests of squamous cell carcinoma (H&E, ×200); B, p16 immunostain with negative staining (×200)

**FIGURE 2:**
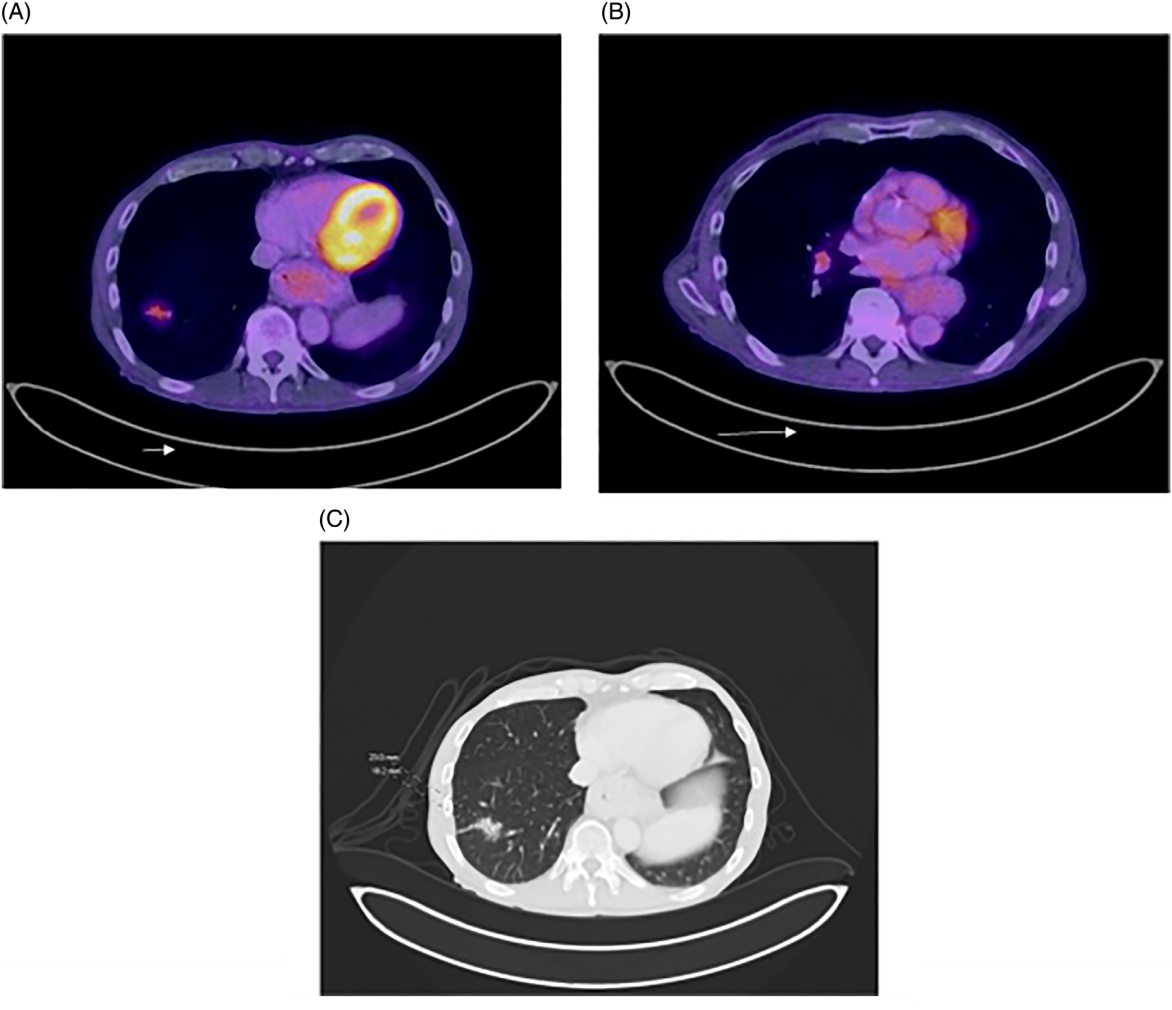
PET/CT showing right lung nodule A, and right hilar lymph node B. CT imaging of the spiculated lung nodule C

A 67‐year‐old male presented with an ulcer in the right ventral tongue, approximately 2 cm in the greatest dimension. He was then found to have SCC of the tongue, which immunohistochemical staining showed to be p16 negative. (Figure 1) He underwent right partial glossectomy with skin graft reconstruction and selective neck dissection, pathology was pT1N2b with negative margins and no extranodal extension. He then received adjuvant radiotherapy. His post‐treatment positron emission tomography (PET) showed a metabolic response in the neck, but a new spiculated right lower lobe mass and hilar and subcarinal nodes were found. (Figure 2) Fine needle aspiration (FNA) from the hilar node showed malignant squamous cells. IHC demonstrated strong staining of the tumor with cytokeratin 5 and p40 and no staining with TTF‐1, napsin A, and p16, Therefore cytology was consistent squamous cell carcinoma, however unable to exclude a second primary right lung SCC with hilar node involvement. Next‐generation sequencing (NGS) with FoundationOne was performed for both primary tongue tumor and hilar lymph node.

Both specimens had the same *TP53* (R273H) and *BRCA2* (W563*) mutations. The specimen from the hilar lymph node had *FAT1* (Q1138*) and *TERT* promoter (−124C > T) mutations in addition to *TP53* and *BRCA2*. *BRCA2* allele frequency was 50% suggesting germline mutation. His family history of cancer included his mother with multiple myeloma and his father with lung cancer and melanoma. So, the patient was referred to genetics and a blood test confirmed germline *BRCA2* mutation. The variants of unknown significance (VUS) were also identical in both specimens. (Table 1) Having the identical *TP53* mutation and VUS in both specimens was consistent with metastatic disease from SCC of the tongue. The patient was treated with carboplatin/5‐Fluorouracil (5‐FU) and cetuximab with initial partial response in lung nodule and hilar lymph node however, he subsequently had progression with bone metastasis. He was briefly on olaparib, and then pembrolizumab without response so he transitioned to hospice.

**TABLE 1:** Genomic alterations and variants of unknown significance found in primary tongue lesion and hilar lymph node

	Tongue lesion	Hilar lymph node
Genomic alteration	***TP53*** R273H ***BRCA2*** W563*	***TP53*** R273H ***BRCA2*** W563* ***FAT1*** Q1138 ***TERT promoter*** −124C > T
Variants of unknown significance	***JUN*** T239I ***MYST3*** I729L ***NOTCH3*** C1222G ***RAF1*** A237G ***SRC*** K106R ***SUFU*** R289Q	***JUN*** T239I ***MYST3*** I1729L ***NOTCH3*** C1222G ***RAF1*** A237G ***SRC*** K106R ***SUFU*** R289Q

## 3 DISCUSSION

**FIGURE 3:**
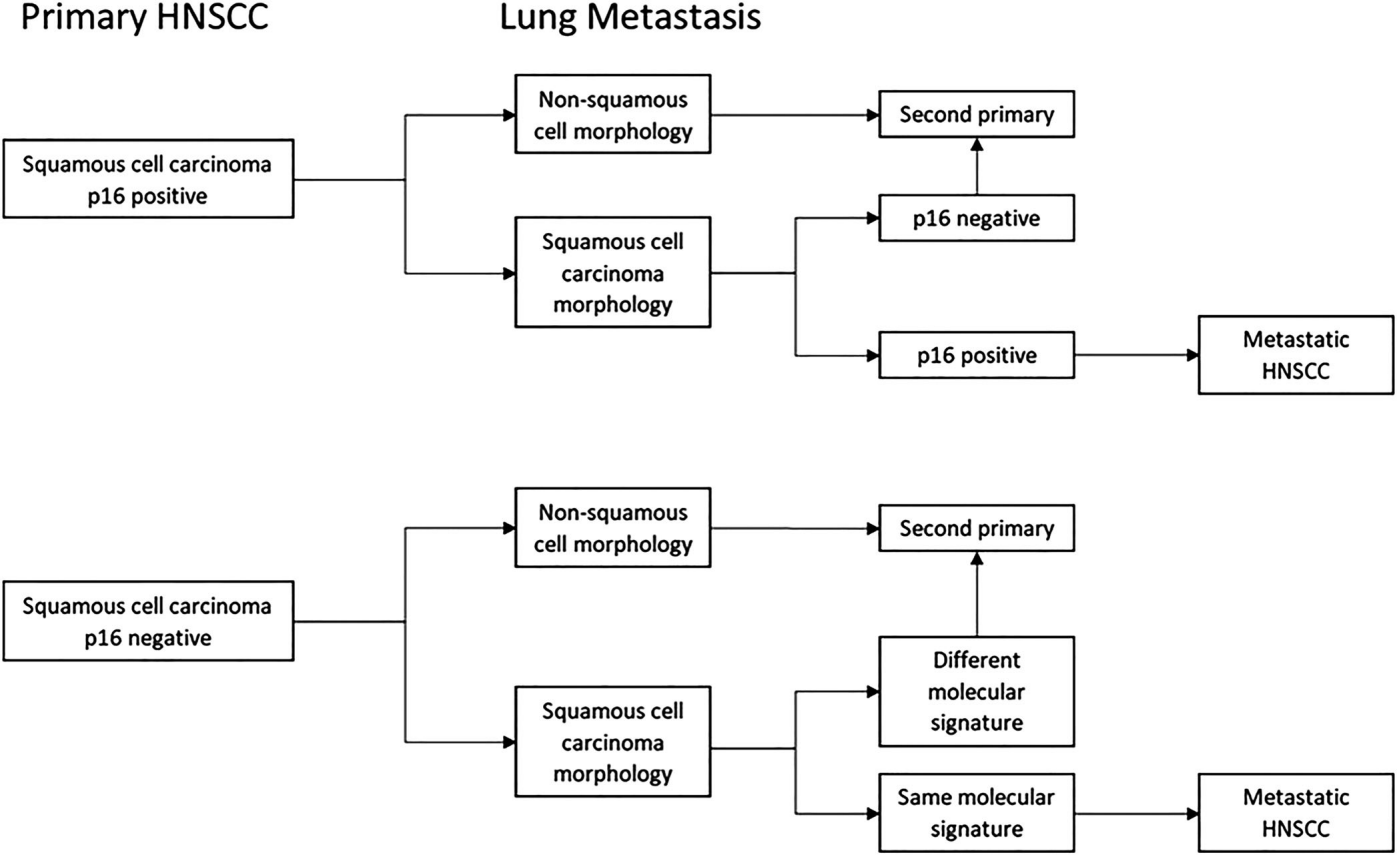
A suggested approach to biopsy from suspicious lung nodule or mediastinal lymph node biopsy in head and neck squamous cell carcinoma

Lung is the most common site of distant metastasis in HNSCC. Given the high incidence of second primary lung squamous cell carcinoma in patients with HNSCC, differentiating the second primary from metastasis helps to identify the next line of treatment. Clinical features can help to guide the treatment. Lung metastasis from HNSCC usually presents as smoothly defined multiple lesions whereas lung cancer is usually a single spiculated lung nodule.^9^ Our patient had a spiculated lung nodule with associated hilar LAD suspicious for second primary lung cancer. On the other hand, his lung finding was seen within 6 months of the initial surgery and he had no tobacco smoking or alcohol history. Recurrence risk in HNSCC is higher in the first year of the initial treatment.^10^ Tobacco smoking increases the risk of the second primary malignancies in HNSCC patients.^11^ However, a recent study using the TP53 mutation status to differentiate metastasis from second primary lung malignancies suggested that 55% of the patients received incorrect treatments based on clinical and immunohistochemical data.^8^ Molecular alterations in pathways affecting the cell cycle, cell death, DNA repair mechanism, and cell differentiation are common in HNSCC.^12^ Incorporation of molecular profiling of the HPV negative HNSCC can help to distinguish second primary lung cancer from metastasis. (Figure 3) Molecular signature with clinical presentation supporting the metastasis from HNSCC, our patient received systemic treatment for metastatic HNSCC.

Interestingly, our patient had *FAT1* and *TERT* promoter mutations in the metastatic tumor, while they were not observed in the primary tumor *FAT1* mutation was observed in 21.7% of the HNSCC samples in The Cancer Genome Atlas (TCGA) database.^12^ FAT1 is known to have a role in cell migration and EMT.^13^, ^14^ *TERT* promoter mutations are common in solid tumors and associated with metastasis and poor survival.^15^, ^16^ Although the tumor heterogeneity could have played a role in the lack of FAT1 and *TERT* promoter mutations in the primary tumor; similar allele frequency with the *TP53* mutation in both samples suggests the same clone with the primary tumor. As *FAT1* and *TERT* are both known to be associated with cell migration and metastasis; the subclones with these two mutations could have been selected following radiation to drive the metastasis. Analysis of larger cohorts to differentiate the molecular characteristics of primary tumor and metastasis for HNSCC might help to identify mechanisms of metastasis.

Somatic mutations of the *BRCA2* gene can be seen in up to 9% of HNSCC.^17^ However, germline mutations can also be identified using NGS.^18^, ^19^ This also brings ethical considerations to share with patients incidental findings from NGS. Our patient knew about the risk of *BRCA2* and hereditary cancer risk and since he had 50% *BRCA2* allele frequency, he consented for genetics counseling which confirmed germline *BRCA2* mutations. Germline *BRCA2* mutation has been identified in SCC of the esophagus and lung.^20^, ^21^ Poly (ADP‐ribose) polymerase (PARP) inhibitors are used in ovarian, breast, and pancreatic cancer patients with germline BRCA mutations.^22^, ^23^, ^24^ However, the role of the PARP inhibitors in other solid tumors with uncommon germline BRCA mutations is not well known. Our patient was briefly on PARP inhibitor without response.

To summarize, the molecular signature of primary HNSCC tumor and hilar node led to three observations in our patient: (a) Molecular signature may help to identify metastasis from second primary lung cancer for p16 negative HNSCC, (b) Further studies needed to identify differences in molecular signatures of primary tumor and metastasis of HNSCC, which may help to better understanding and therapeutics strategies for metastasis, (c) Increased use of NGS may increase identification of germline mutations.

## CONFLICT OF INTEREST

None of the authors have a conflict of interest to declare.

## AUTHOR CONTRIBUTIONS

**Gregory N Gan:** Conceptualization; writing‐review and editing. **Thomas M Schroeder:** Conceptualization; writing‐review and editing. **Andrew Cowan:** Conceptualization; writing‐review and editing. **Nancy Joste:** Conceptualization; data curation; formal analysis; writing‐review and editing.

## ETHICS STATEMENT

The patient was not alive at the time of the manuscript submission and he did not have contact information for next of kin documented in his medical records, therefore we were unable to obtain a written informed consent. This case study was reviewed by the University of New Mexico Human Research Review Committee (HRRC). The HRRC determined that the proposed activity is not research involving human subjects. HRRC review and approval is not required.

## Data Availability

The data that support the findings of this study are available from the corresponding author upon reasonable request.
